# Lung-protective mechanical ventilation for patients undergoing abdominal laparoscopic surgeries: a randomized controlled trial

**DOI:** 10.1186/s12871-021-01318-5

**Published:** 2021-03-30

**Authors:** Trung Kien Nguyen, Viet Luong Nguyen, Truong Giang Nguyen, Duc Hanh Mai, Ngoc Quynh Nguyen, The Anh Vu, Anh Nguyet Le, Quang Huy Nguyen, Chi Tue Nguyen, Dang Thu Nguyen

**Affiliations:** 1 Center of Emergency, Critical Care Medicine and Clinical Toxicology, 103 Military Hospital, Vietnam Military Medical University, 261 Phung Hung road, Ha Dong District, Hanoi City, Vietnam; 2 Critical Care Unit, National Burn Hospital, Vietnam Military Medical University, Hanoi, Vietnam; 3 Department of Cardiothoracic surgery, 103 Military Hospital, Vietnam Military Medical University, Hanoi, Vietnam; 4 Department of Anesthesia and Pain Medicine, 103 Military Hospital, Vietnam Military Medical University, Hanoi, Vietnam; 5 Department of Anesthesia and Pain Medicine, Vietnam National Cancer Hospital, Hanoi, Vietnam; 6 Department of Urology, 103 Military Hospital, Vietnam Military Medical University, Hanoi, Vietnam

**Keywords:** Lung-protective ventilation, Low tidal volume, Recruitment maneuvers, Positive end-expiratory pressure

## Abstract

**Background:**

Pneumoperitoneum and Trendelenburg position in laparoscopic surgeries could contribute to postoperative pulmonary dysfunction. In recent years, intraoperative lung-protective mechanical ventilation (LPV) has been reportedly able to attenuate ventilator-induced lung injuries (VILI). Our objectives were to test the hypothesis that LPV could improve intraoperative oxygenation function, pulmonary mechanics and early postoperative atelectasis in laparoscopic surgeries.

**Methods:**

In this randomized controlled clinical trial, 62 patients indicated for elective abdominal laparoscopic surgeries with an expected duration of greater than 2 h were randomly assigned to receive either lung-protective ventilation (LPV) with a tidal volume (Vt) of 7 ml kg^− 1^ ideal body weight (IBW), 10 cmH_2_O positive end-expiratory pressure (PEEP) combined with regular recruitment maneuvers (RMs) or conventional ventilation (CV) with a Vt of 10 ml kg^− 1^ IBW, 0 cmH_2_O in PEEP and no RMs. The primary endpoints were the changes in the ratio of PaO_2_ to FiO_2_ (P/F). The secondary endpoints were the differences between the two groups in PaO_2_, alveolar-arterial oxygen gradient (A-aO_2_), intraoperative pulmonary mechanics and the incidence of atelectasis detected on chest x-ray on the first postoperative day.

**Results:**

In comparison to CV group, the intraoperative P/F and PaO_2_ in LPV group were significantly higher while the intraoperative A-aO_2_ was clearly lower. C_dyn_ and C_stat_ at all the intraoperative time points in LPV group were significantly higher compared to CV group (*p* < 0.05). There were no differences in the incidence of atelectasis on day one after surgery between the two groups.

**Conclusions:**

Lung protective mechanical ventilation significantly improved intraoperative pulmonary oxygenation function and pulmonary compliance in patients experiencing various abdominal laparoscopic surgeries, but it could not ameliorate early postoperative atelectasis and oxygenation function on the first day after surgery.

**Trial registration:**

https://www.clinicaltrials.gov/identifier: NCT04546932 (09/05/2020).

## Background

Laparoscopy has been widely used in surgical treatment because of its advantages such as minimal invasiveness, better cosmetic outcome, and shorter length of hospital stay. However, the effects of pneumoperitoneum and Trendelenburg position on pulmonary function in laparoscopy have posed particular concerns. Pneumoperitoneum and Trendelenburg position could contribute to atelectasis formation [1], particularly in dependent regions [2] and elevate mechanical stress in pulmonary parenchyma [3], triggering significant perioperative pulmonary dysfunction. In addition, general anesthesia with mechanical ventilation, by decreasing end-expiratory lung volume (EELV) and forming atelectasis, lead to a deterioration in respiratory mechanics and gas exchange [4, 5]. Especially, ventilation patterns with high tidal volumes should overdistend noninjured lungs, thereby activating a local inflammation and coagulation reaction [6, 7].Furthermore, zero-positive end-expiratory pressure or low levels of positive end-expiratory pressure (PEEP) could induce repetitive collapse and reopening of the alveoli, which ultimately result in an inflammatory injury [8].

In recent years, intraoperative lung-protective mechanical ventilation (LPV) has been reportedly able to attenuate ventilator-induced lung injuries (VILI) [9] by employing a low tidal volume (Vt) [10], an appropriate level of PEEP [11], and recruitment maneuvers (RMs) [12]. The goals of these interventions are to minimize alveolar overdistention, to prevent repeated collapse and reopening of alveoli and to reduce atelectasis.

There have been several randomized controlled clinical trials (RCTs) that compared a protective strategy of ventilation with a conventional strategy in various surgical procedures such as cardiac surgery [13], open abdominal surgery [14–17], spinal surgery [12], or thoracic surgery [18, 19]. We tested the hypothesis that the lung-protective ventilation strategy including a low tidal volume, an appropriate level of PEEP and periodic recruitment maneuvers could improve intraoperative oxygenation function, pulmonary mechanics, and early postoperative atelectasis.

## Materials and methods

We performed a randomized controlled trial at Vietnam National Cancer Hospital from January 2020 to July 2020. The trial protocol was approved by the Medical Ethics Committee of Vietnam National Cancer Hospital and Vietnam Military Medical University (QĐ-HVQY 264–2020; chairperson Prof Truong Giang Nguyen; on 10 January 2020). The protocol was also registered in https://www.clinicaltrials.gov/ (Protocol Registration and Results System NCT04546932 on September 5th 2020). Also of important, written informed consent was obtained from all patients before inclusion.

### Participants

Inclusion criteria were those older than 18 years of age, planned to undergo elective abdominal laparoscopic surgeries with an expected duration of greater than 2 h, classified as American Society of Anesthesiologists (ASA) physical status II-III and had a body mass index (BMI) less than 30 kg m^− 2^. Patients were excluded from the study if they met at least one of these following criteria: refusal to participate in the study, preexisting significant cardiac or pulmonary comorbidities (for instance, heart failure, intractable shock, chronic obstructive pulmonary disease, asthma, pulmonary infection, bronchiectasis, pulmonary metastases), preexisting abnormalities on chest X-ray or spirometry, a history of neuromuscular disease, liver cirrhosis (Child B or C), or chronic renal failure with hemodialysis, and the need to continue prolonged mechanical ventilation after surgery.

### Randomization and blinding technique

Participants were randomly assigned to receive either the lung-protective ventilation (LPV group) or the conventional ventilation (CV group) at a ratio of 1:1. The randomization was performed by a physician who did not get involved in the study, using the R program with the “runif”, “as.integer”, “int” and “replace” functions. As a result, a list of random numbers was created in each group. The patients, according to their orders of hospital registration, were numbered and then allocated into the group containing their numbers. The intervention protocols were stored in sealed, opaque numbered envelopes. An anesthesiologist who was not involved in the study opened the envelopes and then set the ventilator in accordance with the protocols in the envelopes. Another anesthetist who was in charge of the patients collected data during surgery. Calculated parameters was processed by the physician responsible for analyzing statistics after data collection. The patients and the surgeons taking part in the procedures were not informed of the ventilator setting. Physicians in post-anesthesia care unit who were not responsible for intraoperative care carried out the postoperative evaluation. The postoperative chest X-ray was analyzed by a radiologist who was not involved in the study.

### Standard procedure

All patients fasted for 12 h before the procedure but were allowed to drink water until 2 h prior to surgery. In the operating room, a radial arterial cannula was inserted to monitor invasive blood pressure, to collect blood gas sample, and to measure the pulse pressure variation (PPV) index in order to guide intraoperative fluid therapy. An epidural catheter was also inserted for postoperative analgesia.

All patients received intravenous fentanyl 2 μg kg^− 1^, lidocaine 40 mg, propofol 2 mg kg^− 1^, and rocuronium 1 mg kg^− 1^, for induction. Anesthesia was maintained using sevoflurane of which the concentration was adjusted to achieve the end-tidal concentration within the range of 1.4–1.8 in oxygen and to keep the PRST score (pressure, rate, sweating, tears) less than 3. If the PRST score was greater than 3, then an additional bolus dose of 0.5 mg kg^− 1^ propofol and 1 μg kg^− 1^ fentanyl was injected along with increasing sevoflurane concentration. On the contrary, if signs of deep anesthesia were presented (PRST score = 0, blood pressure decreased by more than 20% of the baseline values, bradycardia), then the sevoflurane concentration was decreased and 100 ml of ringer lactate solution was rapidly infused within 2 min. If the blood pressure was still lower than 20% of the baseline value in spite of these above-mentioned steps, a bolus dose of 100–200 μg phenylephrine was added. Rocuronium was continuously infused at the rate of 10 μg kg^− 1^ min^− 1^. The solution of bupivacaine 0.1% combined with fentanyl 2 μg ml^− 1^ was infused via the epidural catheter at the rate of 5 ml h^− 1^ after a loading dose of 5 ml prior to skin incision. The pneumoperitoneum was implemented by CO_2_ insufflation at a pressure of 12 mmHg with room temperature in all patients. The intraoperative fluid was managed based on the goal-directed fluid therapy with a crystalloid solution. In brief, no additional fluid was provided if PPV was lower than 10%, otherwise, bolus doses of 250 ml ringer lactate solution were given over 10–15 min. After each bolus dose, PPV was re-assessed and further bolus doses were administered until PPV was lower than 10% [20–22].

Ondansetron 8 mg was injected intravenously 30 min before the end of surgery to prevent postoperative nausea and vomiting. The neuromuscular blockade was reversed using intravenous neostigmine 40–60 μg kg^− 1^ combined with atropine 0.5 mg. Patients were extubated when they met the extubation criteria (spontaneous tidal volume > 6 ml kg^− 1^ and respiratory rate = 12–20 breath min^− 1^, SpO_2_ > 95%, normocarbia, body temperature > 35 °C, positive gag reflexes and ability to follow a verbal command, hemodynamic stability without vasopressor support and ability to lift their heads and hold for 5 s) [23]. Postoperative epidural analgesia for 48 h was conducted using bupivacaine 0.1% combined with fentanyl 2 μg ml^− 1^ at an infusion rate of 5–10 ml h^− 1^ to maintain a visual analogue scale (VAS) score < 3.

### Ventilation protocol

Mechanical ventilation protocol was performed on the anesthesia machine GE healthcare carestation 620. The patient’s ideal body weight was predefined according to these formulas: 45.5 + 0.91 × [height(cm)-152.4] for women or 50 + 0.91 × [height(cm)-152.4] for men. In both two groups, mechanical ventilation was set up at the volume-controlled mode, the inspiration to expiration ratio of 1:2. FiO_2_, after being kept at 1.0 in the induction period, was maintained at 0.4 until extubation. Respiratory rate, starting with 18 breaths min^− 1^, was then modulated to keep the end-tidal carbon dioxide (EtCO_2_) in the normal range of 35–40 mmHg. In CV group, the tidal volume was set at 10 ml kg^− 1^ IBW without PEEP and RM. In contrast, in LPV group, patients were provided with a tidal volume of 7 ml kg^− 1^ IBW and an intraoperative 10 cmH_2_O PEEP. Simultaneously, in LPV group, alveoli were recruited applying a stepwise increase in PEEP (from 4 to 10 cmH_2_O for 3 breaths, 10 to 15 cmH_2_O for 3 breaths, and 15 to 20 cmH_2_O for 10 breaths) with maximum PIP (Peak Inspiratory Pressure) of 50 cmH_2_O [24]. The recruitment maneuvers were performed right after intubation, 30 min after CO_2_ insufflation, then every hour, and finally before extubation. During anesthesia, a plateau pressure of no more than 30 cmH_2_O was targeted in each group.

### Data source and collection

The demographic characteristics including age, gender, height, weight, BMI, ASA physical status and history of coexisting diseases and smoking were recorded. Vital signs (heart rate, blood pressure, SpO_2_, EtCO_2_, core temperature) were also documented every 15 min throughout the surgery. The volume of intravenous fluid (crystalloid, colloid solution), the volume of blood loss and urine output; total given dose of anesthetics, fentanyl, and muscle relaxant, were recorded as well. Arterial blood samples were withdrawn from the radial arterial cannula for blood gas analysis before induction, 1 h after pneumoperitoneum, and day one after operation. The ratio of P/F and the alveolar-arterial oxygen gradient (A-aO_2_) was calculated respectively as P/F = PaO_2_/FiO_2_ and A-aO_2_ = (PB-PH_2_O) × FiO_2_-PaCO_2_/R-PaO_2_ where PB (atmospheric pressure) is 760 mmHg, PH_2_O (saturated vapor pressure at room temperature) is 47 mmHg, and the R (respiration quotient) is 0.8. The dynamic compliance (C_dyn_) was measured directly on the ventilator, and the static compliance (C_stat_) was calculated in accordance with the pre-defined formula as Vt/(plateau pressure– PEEP) with the plateau pressure being measured during the normal ventilation setting using an inspiratory pause at 10% of the inspiratory time. Both types of pulmonary compliance were recorded at H_0_ (after intubation), H_1_ (30 min after pneumoperitoneum), H_2_ (1 h after pneumoperitoneum), H_3_ (2 h after pneumoperitoneum), H_kt_ (10 min after pneumoperitoneum stopped) and H_ro_ (before extubation). Pre- and postoperative (day 1) chest radiography at bedside was obtained and analyzed in a blinded way by a radiologist who was not involved in the study. Pathological chest X-ray was defined as the presence of at least one of the followings: an increase in the thickness of interstitium, atelectasis, pleural effusion, localized or diffuse infiltrates.

### Primary and secondary endpoints

Our hypothesis was that the lung-protective ventilation could improve intraoperative oxygenation function, pulmonary mechanics, and early postoperative atelectasis. The primary endpoints were the intra- and postoperative changes in P/F. The secondary endpoints were the differences between the two groups regarding PaO_2_, A-aO_2_; intraoperative C_dyn_ and C_stat_, and the incidence of atelectasis detected on chest x-ray on the first postoperative day.

### Statistical analysis

The sample size was calculated in accordance with the formula [25]: n = (2 × C)/δ^2^ + 1 with δ = |μ1-μ2|/σ, where n is the sample size in each group, μ1 = mean of P/F in LPV group, μ2 = mean of P/F in CV group, σ is the common standard deviation and c = 7.9 for 80% power. The primary outcomes in the study of Xin Pi (2015) [26] showed that the P/F after 2 h of ventilation in the two groups was 382.21 ± 88.03 and 450.10 ± 70.29 respectively. Replacing μ1 = 382.21, μ2 = 450.10, σ = 88.03 in the formula, n was equal to 27.5 for each group. This represented that the minimum sample size for each group was at least 28 patients.

Statistical analysis was completed using SPSS software version 20.0 (IBM, USA) on an intention-to-treat basis. Whether variables distributed normally or not was tested with the Kolmogorov-Smirnov and Shapiro-Wilk test. Continuous variables, depending on the characteristics of their distribution, were compared applying either Student’s t-test or the Mann-Whitney U test, and consequently were reported as mean ± SD or median and interquartile range (25–75%) as appropriate. Comparisons of normally distributed variables were also performed with one-way ANOVA. As for categorical variables, the χ^2^ test was employed for comparison and the Fisher exact test was used for small frequencies. All the tests were two-tailed, and statistical significance was accepted at *p* < 0.05.

## Results

**Fig. 1:**
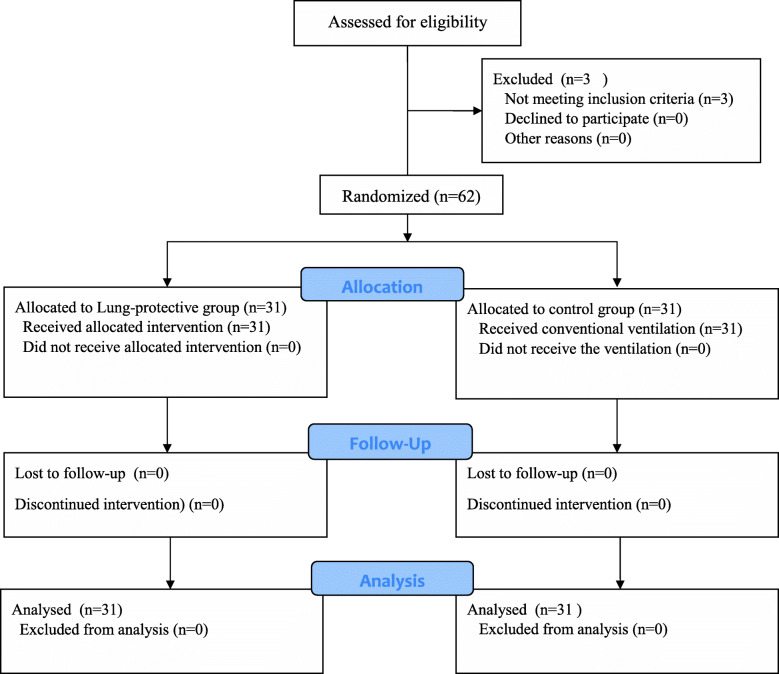
Flow diagram of the process through the phases of the trial

Sixty-five patients were initially assessed for eligibility. Three patients, including two with abnormalities on preoperative chest X-ray and one with a history of COPD, were excluded from the study. Therefore, 62 patients were randomly assigned to the two groups. The enrollment flow diagram is reported in Fig. 1. The demographic characteristics of the participants as well as the surgical and anesthesiological characteristics are presented in Tables 1 and 2, respectively.

**Table 1:** Patients demographic characteristics

	LPV group (***n*** = 31)	CV group (***n*** = 31)
Age (year)	59 ± 9 32–77	55 ± 12 29–74
Height (cm)	162 [159–168]	163 [154–165]
Weight (kg)	53 ± 8 42–68	56 ± 6 40–72
IBW	59 [48–61]	57 [52–64]
BMI (kg m^− 2^)	21 ± 2 18–26	21 ± 3 19–29
Gender (Female/male) (n)	22/9	18/13
ASA (I/II/III) (n)	0/18/13	0/20/11
History of smoking, n (%)	11 (36)	7 (23)
History of hypertension	6 (19)	4 (13)
History of diabetes mellitus	0 (0)	1 (3)

Data are shown as mean ± SD, median [interquartile range] and as percentage as appropriate. *ASA* American Society of Anesthesiologist, *BMI* body mass index, *IBW* Ideal body weight. Differences among groups were not statistically significant

**Table 2:** Surgical and anesthesiological characteristics

		LPV group (***n*** = 31)	CV group (***n*** = 31)
Types of surgery	Gastrectomy	10 (32)	7 (23)
	Colectomy	6 (19)	12 (38)
	Miles’ operation	6 (19)	4 (13)
	LAR surgery	7 (23)	6 (19)
	Others	2 (7)	2 (7)
Duration of mechanical ventilation (minutes)		180 [145–225]	185 [155–220]
Duration of pneumoperitoneum (minutes)		120 [75–140]	105 [80–160]
Intraoperative blood loss (ml)		110 [75–140]	130 [90–160]
Volume of crystalloid given (ml)		700 [525–900]	750 [525–900]
Urine output (ml)		220 [170–220]	200 [150–250]
Total dose of propofol (mg)		100 [100–120]	100 [90–120]
Total dose of fentanyl (μg)		350 [300–350]	350 [300–350]
Total dose of rocuronium (mg)		100 [80–110]	90 [80–110]
Postoperative VAS score		0 [0–1]	0 [0–1]

Data are shown as median [interquartile range] or as percentage as appropriate. *LAR* Low anterior resection. Differences among groups were not statistically significant

### Gas exchange

**Fig. 2:**
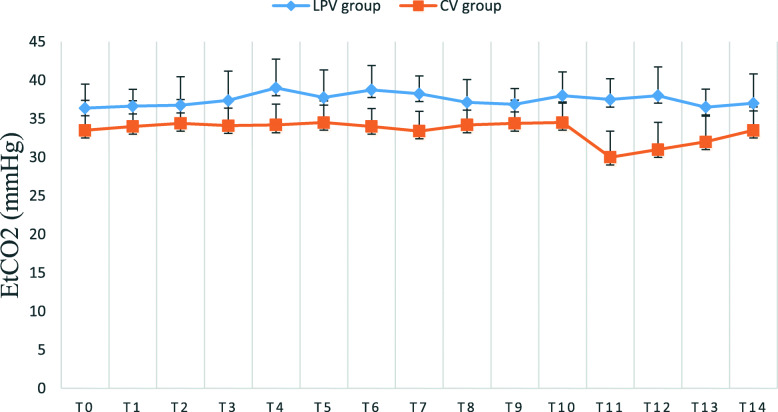
Intraoperative EtCO_2_ in the two groups with interval of 15 min from T_0_ (after intubation) to T_14_ (3.5 h after intubation). Data are reported as mean ± SD. *p* < 0.05 versus CV group

There were no significant differences regarding blood gas between the two groups before and after surgery. The intraoperative PaO_2_ and P/F in LPV group were significantly higher than those in CV group (*p* < 0.05) (Table 3). The intraoperative A-aO_2_ in LPV group was clearly lower than that in CV group, while the PaCO_2_ and EtCO_2_ during surgery in LPV group were higher than those in CV group (*p* < 0.05) (Table 3 and Fig. 2).

**Table 3:** perioperative arterial blood gas analysis

		LPV group (***n*** = 31)	CV group (***n*** = 31)	***p***
PaO2 (mmHg)	Preoperation	86 [78–91]	84 [79–95]	0.2
	1 h after pneumoperitoneum	207 [193–225]	189 [148–206]	**0.001**
	1 day after surgery	98 [81–173]	91 [77–136]	0.4
PaCO2 (mmHg)	Preoperation	35 [34–39]	37 [35–39]	0.07
	1 h after pneumoperitoneum	47 ± 9 36–62	43 ± 6 31–56	**0.03**
	1 day after surgery	38 ± 5 30–49	38 ± 4 31–46	0.7
PH	Preoperation	7.44 [7.42–7.47]	7.44 [7.43–7.45]	0.7
	1 h after pneumoperitoneum	7.37 [7.30–7.40]	7.37 [7.34–7.41]	0.1
	1 day after surgery	7.42 ± 0.03 7.35–7.48	7.41 ± 0.03 7.36–7.46	0.1
P/F	Preoperation	392 ± 58 316–466	405 ± 50 330–473	0.3
	1 h after pneumoperitoneum	518 [483–563]	473 [370–515]	**0.001**
	1 day after surgery	327 [300–524]	319 [285–453]	0.4
A-aO_2_	Preoperation	20 ± 7 9–31	19 ± 7 10–32	0.4
	1 h after pneumoperitoneum	20 ± 15 4–77	55 ± 27 15–106	**< 0.001**
	1 day after surgery	47 ± 34 10–110	57 ± 28 10–105	0.3

Data are shown as mean ± SD or median [interquartile range] as appropriate

### Intraoperative pulmonary mechanics

**Fig. 3:**
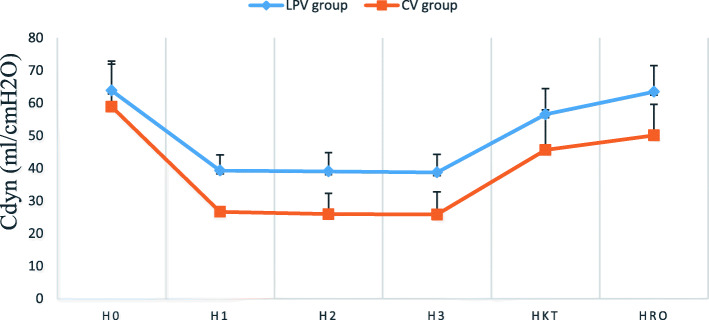
Changes in intraoperative pulmonary dynamic compliance (C_dyn_). Data are reported as mean ± SD. At all time points, the difference between the two groups was significant with *p* < 0.05. H_0_ (after intubation), H_1_ (30 min after pneumoperitoneum), H_2_ (1 h after pneumoperitoneum), H_3_ (2 h after pneumoperitoneum), H_kt_ (10 min after pneumoperitoneum stopped) and H_ro_ (before extubation)

**Fig. 4:**
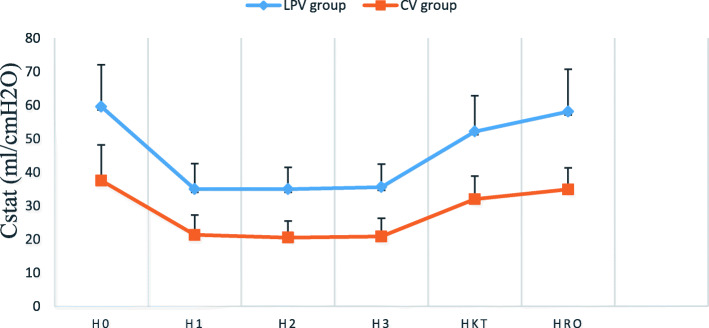
Changes in intraoperative pulmonary static compliance (C_stat_). Data are reported as mean ± SD. At all time points, the difference between the two groups was significant with *p* < 0.05. H_0_ (after intubation), H_1_ (30 min after pneumoperitoneum), H_2_ (1 h after pneumoperitoneum), H_3_ (2 h after pneumoperitoneum), H_kt_ (10 min after pneumoperitoneum stopped) and H_ro_ (before extubation)

**Fig. 5:**
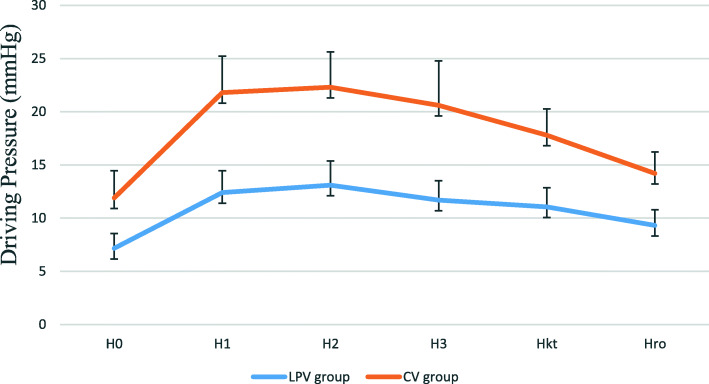
Intraoperative driving pressure (DP) in the two groups. Data are reported as mean ± SD. At all time points, the difference between the two groups was significant with *p* < 0.05. H_0_ (after intubation), H_1_ (30 min after pneumoperitoneum), H_2_ (1 h after pneumoperitoneum), H_3_ (2 h after pneumoperitoneum), H_kt_ (10 min after pneumoperitoneum stopped) and H_ro_ (before extubation)

C_dyn_ and C_stat_ at all the intraoperative time points in LPV group were significantly higher compared to CV group (*p* < 0.05) (Fig. 3 and 4). The driving pressure at all investigated time points in LPV group were substantially lower than those in CV group (*p* < 0.05) (Fig. 5).

### Postoperative observations

There were no differences in the chest X-ray, including the incidence of atelectasis, between the two groups on day one after surgery (Table 4). Length of stay in postoperative care unit was 2 days in both the LPV group and the CV group.

**Table 4:** Pathological chest X-ray test on the postoperative day 1

	LPV group (***n*** = 31)		CV group (***n*** = 31)		***p***
	n	%	n	%	
Normal	21	67.7	20	64.5	0.7
Increased thickness of interstitium	5	16.1	5	16.1	
Atelectasis	0	0	2	6.5	
Diffuse infiltrate	2	6.5	2	6.5	
Localized infiltrate	3	9.7	1	3.2	
Pleural effusion	0	0	1	3.2	

## Discussion

The main findings of this randomized controlled trial were that in comparison to the conventional ventilation, the lung-protective ventilation with a low tidal volume (7 ml kg^− 1^ IBW), 10 cmH_2_O PEEP, and RMs in laparoscopic surgery (1) improved intraoperative oxygenation, (2) increased pulmonary compliance, and reduced driving pressure, (3) did not show beneficial effects on oxygenation or atelectasis formation on the first day after surgery.

Several studies suggest that pulmonary oxygenation function is not significantly affected by abdominal CO_2_ insufflation [27, 28]. However, pneumoperitoneum, especially when prolonged, does predispose patients to decreased arterial oxygenation due to atelectasis and diminished functional residual capacity [29]. With the aim to minimize these consequences, our study show that the LPV improves pulmonary oxygenation during pneumoperitoneum. Explaining our endpoints, intraoperative periodic alveolar recruitment and continuous PEEP – two components of the LPV, has been demonstrated to be effective in improving arterial oxygenation [30, 31] by producing re-expansion and preventing the re-occurrence of atelectasis [29]. These theories were also re-confirmed by Whalen et al. (2006) [32] who, by employing an alveolar recruitment maneuver followed by 12 cmH_2_O PEEP in morbidly obese patients, revealed that this strategy significantly enhanced intraoperative oxygenation. The oxygenation improvement also can be explained by an improvement of the ventilation perfusion matching induced by an appropriate level of PEEP. A previous studies, in which Electrical Impedance Tomograpy (EIT) was used to assess changes in regional ventilation in patients undergoing laparoscopic cholecystectomy, showed that intraoperative PEEP (10cmH_2_O) preserved a more homogeneous ventilation distribution, and hence resulting in a better ventilation perfusion matching as compared to zero PEEP intraoperatively or postoperatively [19].

Alveolar-arterial oxygen gradient (A-aO_2_) has clinical utility as its abnormally high values are associated with shunt, ventilation-perfusion mismatch and gas diffusion abnormalities across the alveolar-capillary membrane. Allen et al. described the A-aO_2_ as a useful tool to evaluate intrapulmonary shunt caused by alveolar collapse [33]. In the present study, A-aO_2_ during pneumoperitoneum, as a marker of shunting, was significantly lower in the LPV group compared to CV group. Similar finding was reported by Jing Liu (2019) [34]. The improved intraoperative oxygenation function in LPV group, however, was at the cost of elevation in PaCO_2_ and EtCO_2_, which may result from the low tidal volume. This hypercapnia seemed not to be harmful to patients since the pH was still kept in a normal range and the elevation of PaCO_2_ was in line with permissive hypercapnia (the rate of increase in PaCO_2_ ≤ 10 mmHg per hour and the upper limit should not higher than 100 mmHg) [35].

Intraoperative pneumoperitoneum and Trendelenburg positioning in laparoscopic surgery have been demonstrated to facilitate atelectasis formation [4, 36, 37] by shifting the diaphragm cranially [27, 38], thereby decreasing pulmonary compliance, leading to collapse of small airways and alveoli [26]. To exemplify this point, Gilda Cinnella (2013) showed that intraoperative pneumoperitoneum and Trendelenburg position worsened respiratory mechanics (increase in lung elastance, static intrinsic PEEP, and total airway resistances) [17]. Dealing with this phenomenon, our study showed that the LPV strategy could partially reverse the deleterious effects of pneumoperitoneum and Trendelenburg position on pulmonary mechanics by decreasing atelectasis and improving dynamic and static compliance before, during, and after pneumoperitoneum. For an explanation of this result, we speculated that the high level of PEEP following the RMs could partially counterbalance the cranial shift of the diaphragm caused by pneumoperitoneum and could induce the corresponding lung expansion. Moreover, PEEP keeps the alveoli open and prevent them from repeated opening and collapse [33], which may, over a long ventilation period, lead to pulmonary injury [26]. However, the use of a high level of PEEP intraoperatively posed a significant concern about barotrauma in normal lung and hence may be associated with lung injuries [39]. The level of PEEP in our study should be well tolerated since the driving pressure in LPV group was kept quite low (< 15 cmH_2_O) (Fig. 5) and significantly lower than those in CV group. A cohort study in 2019, in which 1913 patients undergoing cardiac surgery were provided with a protective ventilation bundle comprising of Vt < 8 ml/kg IBW, modified driving pressure < 16 cmH_2_O, and PEEP ≥5 cmH_2_O, revealed that the modified driving pressure was independently associated with decreased PPCs (OR 0.51, 95% CI, 0.39 to 0.66), but Vt < 8 ml/kg and PEEP ≥5 cmH_2_O were not [40].

Atelectasis develops in as much as 90% of patients undergoing general anesthesia [41] and can persist to different degrees after surgery. The etiologies of atelectasis formation during intraoperative short-term ventilation in normal lungs are the compression of lung parenchyma [42], reabsorption of intraalveolar gas [43, 44], collapse of small airways [45], and surfactant dysfunction. The unventilated lung areas are more likely to occur near to the diaphragm with an estimated incidence of 3 to 6% [46, 47] to 20 to 25% [41]. The incidence of atelectasis in our study was fairly low (2 patients occupied 6.5% in CV group) and there was no significant difference between the two groups. One of the limitations in our study was that chest x-ray is much less sensible as compared to CT scanner with regard to atelectatic detection. For this reason, we could not identify pentients with low levels of atelectasis.

There were some limitations to our study. Firstly, our trial considered intraoperative protective mechanical ventilation using bundles of interventions which included a low tidal volume, a high level of PEEP, accompanied by periodic lung recruitment maneuvers. It is difficult to determine the role of each component and to conclude which provided the benefits: the decrease in tidal volume, or the high level of PEEP or the recruitment maneuvers or both. Moreover, to what extent low tidal volumes succeeded in preventing barotrauma and volutrauma could not be analyzed. Secondly, the degree of PEEP should be titrated individually. Several factors that affect individual titration of PEEP during general anesthesia are (1) the respiratory system mechanics [17], (2) oxygenation target [12], (3) level of EELV [48], and (4) distribution of ventilation using electric impedance tomography [19, 49]. Thus, 10 cmH_2_O PEEP in our study may not be suitable for all patients. Thirdly, compared with computed tomography, chest X-ray reportedly underestimate the occurrence of atelectasis and pulmonary morphological alterations [50].

## Conclusion

Lung-protective mechanical ventilation consisting of a low tidal volume of 7 ml kg^− 1^ IBW, 10 cmH_2_O PEEP and regular recruitment maneuvers in abdominal laparoscopic surgeries can significantly improve intraoperative oxygenation function, pulmonary compliance, but can not prevent early postoperative atelectasis formation on the first day after surgery. Larger sample size and long-term evaluation are recommended for future studies.

## Data Availability

The datasets used and/or analyzed during this study are available from the corresponding author on reasonable request.

## Abbreviations

LPV: Lung-protective ventilation
VILI: Ventilator-induced lung injury
Vt: Tidal volume
PEEP: Positive end-expiratory pressure
RMs: Recruitment maneuvers
IBW: Ideal body weight
CV: Conventional ventilation
C_dyn_: Dynamic compliance
C_stat_: Static compliance
P/F: The ratio of PaO_2_ to FiO_2_
A-aO_2_: Alveolar-arterial oxygen gradient
mCPIS: Modified clinical pulmonary infection score
PPCs: Postoperative pulmonary complications
EELV: End-expiratory lung volume
ASA: American society of anesthesiologists
BMI: Body mass index
PPV: Pulse pressure variation
VAS: Visual analogue scale
EtCO_2_: End-tidal carbon dioxide
PRST: Pressure, rate, sweating, tears
